# Supplementary material for: A diminutive new basilosaurid whale reveals the trajectory of the cetacean life histories during the Eocene
Source: Commun Biol. 2023 Aug 10;6:707. doi: 10.1038/s42003-023-04986-w (PMC10415296; doi:10.1038/s42003-023-04986-w)
Supplement: Supplementary file 6 — Supplementary Data 3 [file 42003_2023_4986_MOESM6_ESM.zip › Supplementary Data 3/Supplementary Data 1_BTD_ASR/trait_0186_tree.plot.pdf]

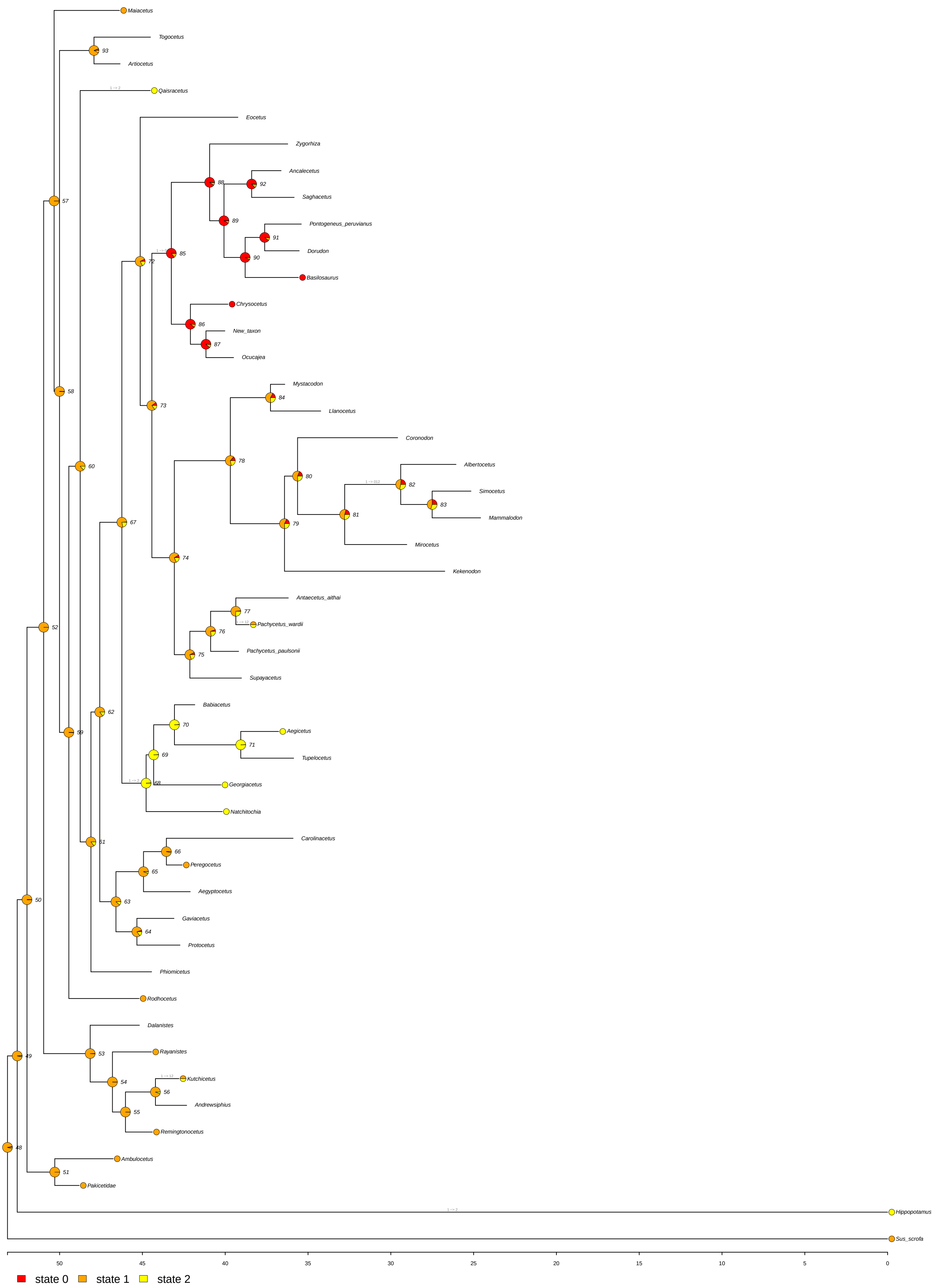

Maiacetus
Togocetus
93
Artiocetus
Qaisracetus
Eocetus
Zygorhiza
Ancalecetus
88
92
Saghacetus
89
Pontogeneus_peruvianus
91
Dorudon
90
85
Basilosaurus
72
Chrysocetus
86
New_taxon
87
Ocucajea
Mystacodon
84
Llanocetus
Coronodon
78
Albertocetus
80
82
Simocetus
83
Mammalodon
81
79
Mirocetus
74
Kekenodon
Antaecetus_aithai
77
Pachycetus_wardii
76
Pachycetus_paulsonii
75
Supayacetus
Babiacetus
70
Aegicetus
71
Tupelocetus
69
68
Georgiacetus
Natchitochia
Carolinacetus
66
Peregocetus
65
Aegyptocetus
63
Gaviacetus
64
Protocetus
Phiomicetus
Rodhocetus
Dalanistes
Rayanistes
53
54
Kutchicetus
56
Andrewsiphius
55
Remingtonocetus
48
Ambulocetus
51
Pakicetidae
Hippopotamus
Sus_scrofa
57
58
60
67
73
62
59
61
52
50
49
50 45 40 35 30 25 20 15 10 5 0
state 0
state 1
state 2
